# Spatial and sex‐specific hippocampal proteomic analysis of an early Alzheimer’s disease mouse model using MALDI‐imaging

**DOI:** 10.1002/alz.086989

**Published:** 2025-01-09

**Authors:** Ana Contreras, Raquel Jimenez‐Herrera, Souhail Djebari, Jaime Mulero‐Franco, Juan D. Navarro‐López, Lydia Jimenez‐Diaz

**Affiliations:** ^1^ Neurophysiology & Behaviour Lab, University of Castilla‐La Mancha, Ciudad Real Spain

## Abstract

**Background:**

A key neuropathological feature in the early stages of Alzheimer's disease (AD) involves hippocampal dysfunction arising from the accumulation of amyloid‐β (Aβ). Previously, our laboratory identified a shift in the synaptic plasticity long term potentiation (LTP)/long term depression (LTD) induction threshold, leading to memory deficits in a non‐transgenic murine model of early AD generated by intracerebroventricular (icv.) injections Aβ_1‐42_ oligomers (oAβ_1‐42_), one of the most predominant pathogenetic factors in initial stages of the disease. However, the specific alterations in proteins within hippocampal subfields remain uninvestigated. Here, we hypothesized that there might be unique protein changes in the hippocampus induced by oAβ_1‐42_ and potentially influenced by sex‐dependent factors.

**Method:**

To do so, we evaluated hippocampal‐dependent memory using a Barnes maze and the Object location memory test (OLM) in the non‐transgenic model of early AD. Both memory encoding and retrieval deficits were shown 1 hour post‐icv. injection, although no significant sex differences were found. Then, an innovative spatial proteomic study was performed to assess the hippocampal proteome using matrix‐assisted laser desorption/ionization (MALDI) imaging mass spectrometry that allows peptide detection with spatial resolution directly on tissue sections.

**Result:**

Results from sixteen brains from C57BL/6 adult male and female mice 1 hour post‐icv. injected with oAβ_1‐42_ or vehicle were analyzed. MALDI imaging was performed using a RapifleXTM MALDI TissuetyperTM TOF/TOF mass spectrometer followed by protein identification by traditional MS/MS directly on the tissue. To precisely delineate anatomical hippocampal region and subfields, a Nissl staining was performed at adjacent tissue sections. More than 600 peptides were detected, and several of them showed significant differences in expression levels due to treatment or sex. To further analyze the data, the protein‐protein interaction (PPI) between the identified changed proteins was analyzed using the STRING database.

**Conclusion:**

The specific hippocampal subfields changes in the proteome caused by early amyloidosis provide new insight in the pathogenesis of AD and valuable potential biomarkers for early diagnosis and potential therapies.

**Acknowledgements**: Grants PID2020‐115823‐GB100/ MCIN/AEI/10.13039/501100011033 and SBPLY/21/180501/000150/ JCCM and “ERDF A way of making Europe” both to LJ‐D and JDN‐L. Margarita Salas Postdoctoral Research Fellow to AC funded by European Union NextGenerationEU/PRTR.

